# MicroRNA Profiling in Human Neutrophils during Bone Marrow Granulopoiesis and In Vivo Exudation

**DOI:** 10.1371/journal.pone.0058454

**Published:** 2013-03-12

**Authors:** Maria T. Larsen, Christoffer Hother, Mattias Häger, Corinna C. Pedersen, Kim Theilgaard-Mönch, Niels Borregaard, Jack B. Cowland

**Affiliations:** 1 The Granulocyte Research Laboratory, Department of Hematology, National University Hospital, University of Copenhagen, Copenhagen, Denmark; 2 The Epigenome Research Laboratory, Department of Hematology, National University Hospital, University of Copenhagen, Copenhagen, Denmark; NIAID, United States of America

## Abstract

The purpose of this study was to describe the microRNA (miRNA) expression profiles of neutrophils and their precursors from the initiation of granulopoiesis in the bone marrow to extravasation and accumulation in skin windows. We analyzed three different cell populations from human bone marrow, polymorphonuclear neutrophil (PMNs) from peripheral blood, and extravasated PMNs from skin windows using the Affymetrix 2.0 platform. Our data reveal 135 miRNAs differentially regulated during bone marrow granulopoiesis. The majority is differentially regulated between the myeloblast/promyelocyte (MB/PM) and myelocyte/metamyelocyte (MC/MM) stages of development. These 135 miRNAs were divided into six clusters according to the pattern of their expression. Several miRNAs demonstrate a pronounced increase or reduction at the transition between MB/PM and MC/MM, which is associated with cell cycle arrest and the initiation of terminal differentiation. Seven miRNAs are differentially up-regulated between peripheral blood PMNs and extravasated PMNs and only one of these (miR-132) is also differentially regulated during granulopoiesis. The study indicates that several different miRNAs participate in the regulation of normal granulopoiesis and that miRNAs might also regulate activities of extravasated neutrophils. The data present the miRNA profiles during the development and activation of the neutrophil granulocyte in healthy humans and thus serves as a reference for further research of normal and malignant granulocytic development.

## Introduction

Differentiation of the PMN is a tightly regulated process in which the myeloid progenitor cells, the myeloblasts (MBs), divide and mature along a well defined path in the bone marrow (granulopoiesis). The different stages of maturation are characterized by distinct morphological features such as cell size, nuclear shape, and granular content [1]. Promyelocytes (PM) and myelocytes (MC) are also capable of undergoing cell division and cell cycle arrest occurs between the myelocyte and metamyelocyte (MM) stages [2]–[4]. Terminal differentiation proceeds through the band cell (BC) and segmented cell (SC) stages ending with the release of mature PMNs to peripheral blood [1].

We have previously described the *in vivo* mRNA and protein profiles of granule proteins [5], transcription factors [6], and cell-cycle regulatory proteins [2] during neutrophil development in the bone marrow. Those studies were based on neutrophil precursors isolated at different stages of maturation from normal human bone marrow. Furthermore, cells purified by this technique have been used for a global description of mRNA expression profiles during granulopoiesis by mRNA microarray analysis [3]. We have also collected peripheral blood PMNs and PMNs from skin window chambers and examined the changes in their transcription profile by mRNA microarray analysis [7].

The main function of neutrophils is to detect and destroy invading microorganisms in tissues [8]. This involves attachment to the blood vessel wall at sites of inflammation, activation of the PMNs, mobilization of their secretory vesicles, and up-regulation of extracellular adhesion molecules [8]. Attachment to the blood vessel wall and subsequent extravasation are succeeded by migration through tissue to the inflammatory focus. During this process, PMNs are exposed to chemotactic stimuli, inflammatory cytokines, and anti-apoptotic growth factors which influence the gene expression profile of the cells [9]–[11].

The temporal expression of the genes encoding granule proteins, which can be divided into those stored in azurophil granules (formed in MB&PMs), in specific granules (formed in MC&MMs), and in gelatinase granules (formed in BCs) [8], is tightly regulated during granulopoiesis in the bone marrow by the specific up- and down-regulation of essential transcription factors such as RUNX1, PU1, C/EBP-α, and C/EBP-ε [6], [12]. C/EBP-α and C/EBP-ε have furthermore been shown to be important for cell cycle arrest and the initiation of terminal differentiation, underscoring the importance of a strictly regulated temporal expression of transcription factors for correct neutrophil development. Different factors play a role in the fine-tuning of transcription factor expression and miRNAs are potential candidates by virtue of their ability to regulate protein synthesis. Regulation of protein expression by miRNAs may also be essential in neutrophils which have migrated to an inflammatory site in order to adapt to their new environment.

MiRNAs are a group of small non-coding RNAs (19–23 nt) that mainly regulate protein expression post-transcriptionally by binding to complementary sequences of the 3′ UTR of mRNAs thereby destabilizing the mRNA or inhibiting its translation. A regulatory role for miRNAs has been demonstrated for several biological processes, such as proliferation, differentiation, and apoptosis, and the dysregulation of miRNA expression has been shown to contribute to the development and progression of cancer [13]–[16]. This also applies to the development of neoplastic myeloid diseases such as myelodysplastic syndrome (MDS) and acute myeloid leukemia (AML) [17]–[19] and points to an important role of miRNAs as regulators of normal granulopoiesis.

The purpose of this study was to determine the miRNA profile during normal human granulopoiesis starting with the first identifiable granulocytic precursor cell in the bone marrow (the myeloblast) and ending with activated neutrophils that have migrated into the tissue. We found 135 differentially expressed miRNAs during neutrophil development in the bone marrow. These could be divided into six clusters according to their expression pattern. In addition, we found seven miRNAs that were up-regulated in neutrophils that had migrated into the tissue compared to neutrophils in peripheral blood. The expression patterns of the miRNAs were verified by quantification of one or two representative miRNAs from each cluster using real-time PCR.

## Materials and Methods

### Ethics Statement

All blood and bone marrow donors as well as participants in skin window experiments gave written informed consent before giving donations according to the guidelines established by the local Ethics Committee of the Cities of Copenhagen and Frederiksberg. The study was approved by the local Ethics Committee (H-1-2011-165).

### Isolation of Neutrophil Precursors from Bone Marrow and PMNs from Peripheral Blood

Neutrophils and their precursors were isolated as described previously from bone marrow and peripheral blood drawn from healthy volunteers [6]. Three donors were used for array analysis (two females and one male, aged between 26 and 28 years) and five donors were used for RT-PCR verifications (one female and four males, aged between 23 and 32 years).

### Collection and Purification of PMNs from Peripheral Blood and Skin Lesions

PMNs and skin window samples were collected in parallel from three healthy donors and purified using immunomagnetic depletion of non-granulocytic cells as previously described (all three donors were males, aged 37, 47 and 56).

### Cytospins

1–2×10^5^ cells were added to a cytospin column followed by centrifugation (400 g for 10 min). The cytospins were stained with May-Grünwald-Giemsa.

### RNA Isolation and Quantitative Real-time PCR

Total RNA was prepared with Trizol® (Invitrogen, Carlsbad, CA, USA) according to the manufacturer’s recommendations and the concentration was determined by measurement on a Nanodrop® 2000 Spectrophotometer (Thermo Scientific, Waltham, MA, USA).

### Quantitative Real-time PCR

#### mRNA targets

For reverse transcription of RNA to first-strand cDNA, 1 µg RNA was diluted in 10 µl DEPC-treated water. 1 µl 50 µM random hexamer primers (Invitrogen) was added. The solution was denatured at 70°C for 10 min followed by the addition of 4 µl 5× First Strand Buffer (Promega), 1 µl 10 mM dNTP, 1 µl RNAsin (Invitrogen), 2 µl 0.1 M DTT (Promega), and 1 µl Superscript II Reverse Transcriptase (Invitrogen). The mixture was incubated at 22°C for 10 min, 45°C for 45 min, and 98°C for 5 min.

Quantitative PCR analysis was performed on an MX 3000 P real-time PCR system (Stratagene, La Jolla, CA, USA). The cDNA was diluted 1∶25 in DEPC-treated water and 5 µl were mixed with 5 µl TaqMan® Universal PCR Master Mix (Applied Biosystems, Foster City, CA, USA) and 0.5 µl of 20× Assay-on-Demand gene expression Master Mix (Applied Biosystems) for *β-actin*: Hs99999903_m1 and one of the following mRNAs: myeloperoxidase *(MPO)*: Hs00165162_m1, *Lactoferrin (LTF)*: Hs00158924_m1, N-formyl-methionyl-leucyl-phenylalanine receptor *(fMLP-R)*: Hs00181830_m1, *IL-8*: HS00174103_m1, or *SERPINA1*: Hs00165475_m1 (Applied Biosystems). The samples were amplified using the following conditions: 50°C for 2 min, 95°C for 10 min, and 40 cycles of 95°C for 15 sec and 60°C for 1 min. β-actin was used as an internal control for normalization and all samples were run in triplicates.

#### miRNA targets

For the reverse transcription of miRNA to first strand cDNA, 10 ng RNA in DEPC-treated water were denatured at 85°C for 2 min and added to a mastermix consisting of 0.15 µl dNTP mix, 1.5 µl RT buffer, 1 µl Multiscribe RT enzyme, 0.19 µl RNase inhibitor and 4.16 µl RNase free water (All Taqman miRNA reverse transcription kit, Applied Biosystems). Thereafter, 1.5 µl of one of the following Taqman miRNA assays was added to the mastermix: miR-130a-3p (000454), miR-27a-3p (000408), miR-126-3p (000450), miR-155 (002633), miR-146a (000468), miR-183-5p (000484), miR-99b (000436), miR-34c-3p (241009_mat), miR-223-5p (002098), miR-26a (00405), miR-212 (00515), miR-132* (002132) (Applied Biosystems) along with 1.5 µl of the primer for RNU6B (001093). Reaction mixtures were incubated at 16°C for 30 min, 42°C for 30 min, and 85°C for 5 min.

Quantitative PCR analysis was performed on an MX 3000 P real-time PCR system (Stratagene). cDNA was diluted 1∶2,33 and 3 µl cDNA were mixed with 5 µl 2× Universal Master Mix without Emperase UNG, 3.84 µl DEPC-treated water, and 0.5 µl 20× TaqMan MicroRNA assay for miR-130a-3p (000454), miR-27a-3p (000408), miR-126-3p (000450), miR155 (002633), miR-146a (000468), miR-183-5p (000484), miR-99b (000436), miR-34c-3p (241009-mat) miR-223-5p (002098), miR-26a (000405), miR-212 (000515), miR-132* (002132), or RNU6B(001093) (Applied biosystems). The samples were amplified using the following conditions: 95°C for 10 min, 40 cycles of 95°C for 15 sec. followed by 60°C for 1 min. RNU6B was used as an internal control and all samples were run in triplicate.

### Microarray Labeling, Hybridization, and Statistic Analysis

RNA from the four populations of cells from each of the three healthy donors and from PMNs and extravasated PMNs in skin window from three other healthy donors, was used to generate biotinylated cRNA. Biotinylated cRNA was labeled using FlashTag™ Biotin HSR RNA Labeling Kit and hybridized to a GeneChip miRNA 2.0 Array (Affymetrix Inc, Santa Clara, CA, USA) containing 1105 distinct microRNAs based on Sanger miRBase Release 15.0. The raw data in the Affymetrix CEL files were normalized by the RMA method (robust multiarray analysis). One-way analysis of variance with moderated F-statistics (LIMMA) was performed to identify differentially expressed miRNA genes between the MB/PM, MC/MM, BC/SC, and PMN populations from the 3 donors and between the PMN population and skin window PMNs from three other donors. The raw p-values were adjusted by the Benjamini-Hochberg method. For cluster analysis, distances between genes were calculated using Pearson’s correlation. Hierarchical cluster analysis (hclust) was applied to determine cluster affiliation of the different miRNAs selected. miRNA targets for miRNAs annotated in miRBase were predicted using the prediction programs MiRanda and TargetScan.

3′ UTR analysis was performed using the prediction programs DIANAmt, miRanda, miRDB, miRwalk, PICTAR4, PICTAR5, and TargetScan. Only miRNAs with a target in a given 3′ UTR predicted by four or more algorithms and with a minimum of seven nucleotides in the seed sequence were selected for further analysis.

### Statistic Analysis on Real Time PCR Verification Experiments

To determine significance between different developmental stages and conditions, comparisons were made using the Student t test. For all statistical tests, p-values <0.05 were considered statistically significant.

### FACS Analysis

For determination of cellular purity, 5 µl of the PE-conjugated antibodies (IgG_1,_ BD Pharmigen, San Jose, CA, USA), CD5 (R0842 DAKO, Glostrup, DK), CD10 (555375 BD Pharmigen), CD14 (R0864 DAKO), CD19 (562321 BD Bioscience), CD56 (555516 BD Bioscience), CD61 (555754 BD Pharmingen), and GlyA (555570 BD Pharmingen), respectively, were used for labeling of the MB/PM and the MC/MM populations. The same antibodies and CD49d (555503 BD Pharmingen) were used for the BC/SC population while antibodies against CD10, CD14, and CD49d were used in the PMN population. For each reaction 1–2×10^5^ cells were incubated with each antibody on ice for 15 min and then washed twice followed by centrifugation (500 g for 4 min). The cells were resuspended in 150 µl 0.1% PFA in PBS and analyzed by flow cytometry using a FACS-Calibur (BD bioscience) and evaluated using CellQuest software.

## Results

### Purification of Neutrophil Precursors from Bone Marrow and Mature Neutrophils from Peripheral Blood

**Figure 1:**
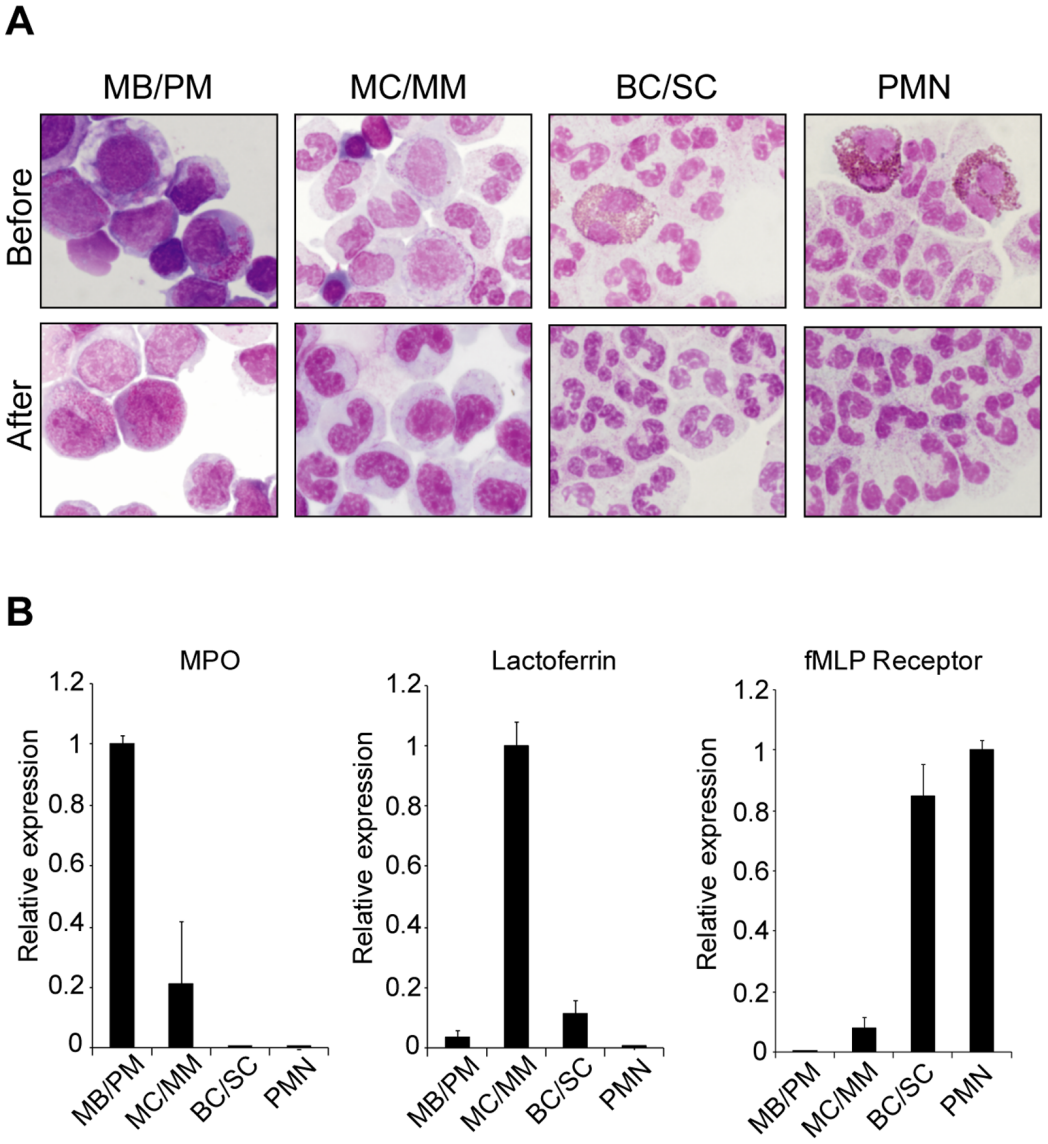
Purification of human granulocytes and precursors. A, Cytospins (1–2×10^5^ cells) of the MB/PM, MC/MM, BC/SC, and PMNs populations from one representative donor before and after immunomagnetic depletion of non-neutrophil cells. B, Real-time PCR data on RNA purified from the four populations in three donors. The expression of the marker mRNAs is shown relative to the cell population with the highest amount, which is given the value 1. MPO expression peak in the MB/PM population, Lactoferrin in the MC/MM population, and high expression of the fMLP-receptor is seen in the BC/SC and PMN populations. Error bars (standard deviation, SD) show the difference in expression between the three donors.

Three cell populations consisting of MB/PMs, MC/MMs, and BC/SCs, respectively, were purified from human bone marrow by Percoll density centrifugation followed by immunomagnetic depletion of non-neutrophil cells. PMNs from peripheral blood were purified by density centrifugation and immunomagnetic purification as described previously [6]. The purity of neutrophil precursors and PMNs regarding other types of blood cells was assessed by flow cytometry as described previously [3], [6]. A proper separation of the bone marrow-derived neutrophils was assured by morphological examination of cytospins of the three cell populations (Fig. 1A) and by real-time PCR analysis of gene markers specifically expressed in MB/PMs (*MPO*), MC/MMs (*LTF*), and BC/SCs (*fMLP-R*) (Fig. 1B) [6].

### miRNA Expression Profiles during Granulopoiesis

RNA purified from MB/PMs, MC/MMs, BC/SCs, and PMNs from three healthy donors was analyzed for miRNA expression by hybridization to miRNA arrays. We identified 135 miRNAs that were differentially expressed when comparing the four cell populations by one-way ANOVA analysis (adjusted p-value <0.01). Hierarchical cluster analysis (hclust) was applied to determine cluster affiliation of the different miRNAs. The classical multi-dimensional scaling plot (MDS-plot) illustrates the dissimilarities between the miRNA profiles for the four populations of cells (Fig. S1). The BC/SC and PMN populations are very close in the MDS plot, which reflects the high similarity in the miRNA profiles of these two cell populations.

**Figure 2:**
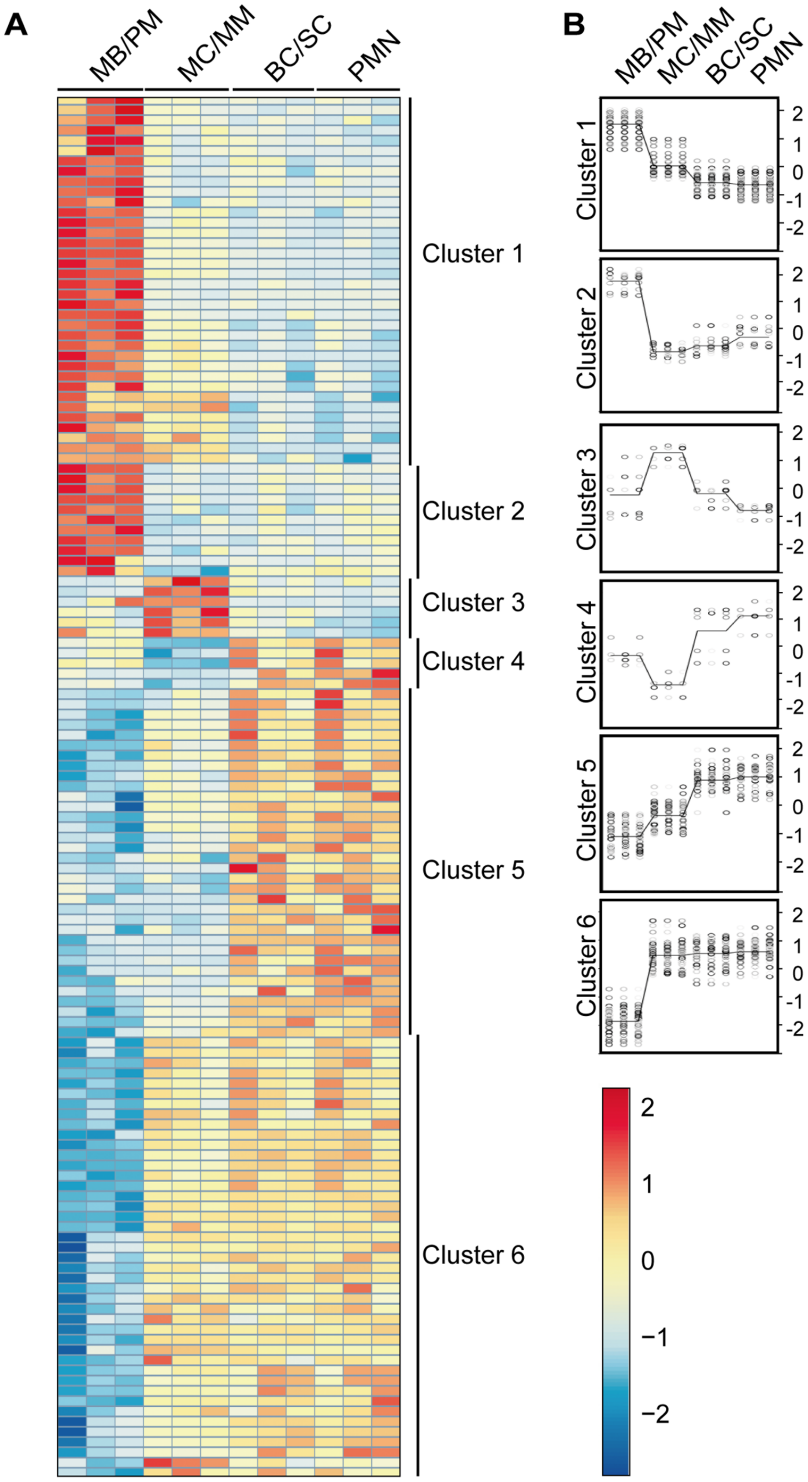
miRNA array analysis of mature human granulocytes and precursors. A, Heatmap illustrating the different miRNA expression profiles for the 135 differentially regulated miRNAs in bone marrow and peripheral blood (red indicates high expression and blue low expression). Each brick row downwards represents one donor. The four different cell populations are marked on the top of the heatmap and the cluster classification is shown on the right side. B, Using hierarchical cluster analysis (hclust) the cluster affiliation of the different miRNAs was determined showing the different expression profiles on a Log2 scale. Each bullet indicates the expression level of a single miRNA in one donor and the line represents the average expression.

The 135 differentially expressed miRNAs were divided into six clusters as illustrated in the heat map in figure 2. The miRNAs in the different clusters are listed in table S1. The miRNAs in cluster 1 (36 miRNAs) showed high expression in the MB/PM population followed by a steep decline at the transition to the MC/MM population and with a slight further decrease in expression with maturation. The miRNAs in cluster 2 (11 miRNAs) showed an even more significant decrease in expression at the transition between MB/PMs and MC/MMs and then a minor increase in expression during the following maturation steps. Expression of the miRNAs in cluster 3 (6 miRNAs) peaked in the MC/MM population and then returned to a lower expression in the more mature stages of the cells. The miRNAs in cluster 4 (5 miRNAs) had the opposite expression pattern with an initial high miRNA expression, then a sharp decline in the MC/MM population, and then again a steep increase in expression in the more mature stages of the cells. The cluster 5 population (34 miRNAs) had a miRNA expression profile with low expression in the MB/PM population followed by a stepwise increase in expression from the MC/MM population to the PMN population. The miRNAs in cluster 6 (43 miRNAs) showed initial low expression like the cluster 5 miRNAs, but in this case, a steep increase in the expression occurred at the transition to the MC/MM population, which remained high during maturation.

We analyzed the data to see how the fold change (FC) between the different populations is distributed (adjusted p-value <0.05) and found that the majority of miRNAs (119) are differentially regulated either upwards or downwards between the MB/PM and MC/MM populations (Fig. S2). Only 18 miRNAs had a significant change between the MC/MM and the BC/SC populations. 16 miRNAs showed a significant change both between the MB/PM and MC/MM populations and the MC/MM and BC/SC populations.

No significant change was seen between the BC/SC and the PMN populations. This indicates that the difference between these two cell populations is very small with regard to the miRNA expression profiles as shown in the MDS plot (Fig. S1).

### Purification of PMNs from Peripheral Blood and Exudated Neutrophils from Skin Windows

**Figure 3:**
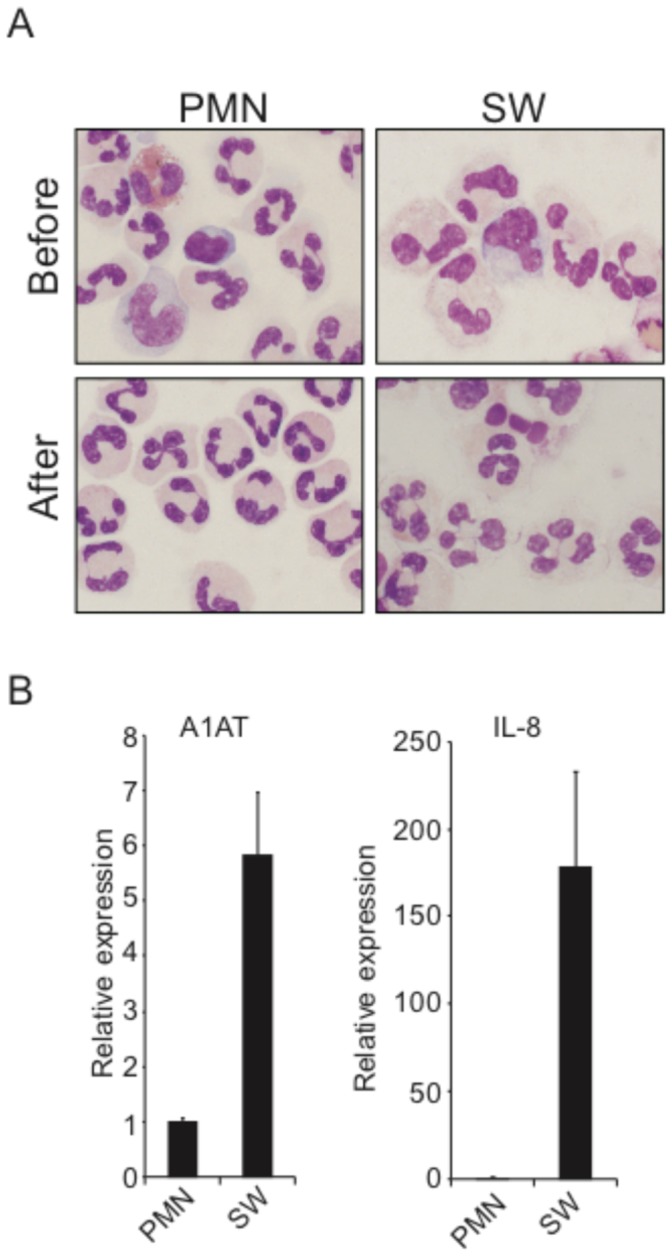
Purification of human neutrophils from skin window. A, Cytospins (1–2×10^5^ cells) of the PMN and skin window (SW) neutrophil populations before and after immunomagnetic depletion of non-neutrophil cells. B, RT-PCR data on RNA purified from the two different cell populations. Expression of the marker mRNAs IL-8 and A1AT are shown relative to the cell population with the lowest amount, which is given the value 1. Error bars (SD) show the difference in expression between two donors.

To investigate the changes in the miRNA profile of neutrophils migrated to skin wounds, RNA was isolated from peripheral blood PMNs and activated neutrophils in skin windows from three different donors. The PMN populations were purified from peripheral blood using Lymphoprep followed by immunomagnetic depletion of non-neutrophil cells. The neutrophils migrated to skin windows were collected in plasma and likewise subjected to immunomagnetic depletion of non-neutrophil cells. The purity of the neutrophil precursors and PMNs regarding other types of blood cells was testified by non detectable levels of non-neutrophil transcripts measured on the mRNA microarray [7] and further evaluated by morphological examination of cytospins of the two cell populations (Fig. 3A). Real-time PCR analysis of gene markers up-regulated in PMNs migrated to the skin window (interleukin 8 (IL-8) and α1-antitrypsin (A1AT)) compared to PMNs from peripheral blood was performed to confirm a difference in gene expression between the two cell populations (Fig. 3B) [7], [20].

### miRNA Expression Profiles in Exudated Neutrophils

**Figure 4:**
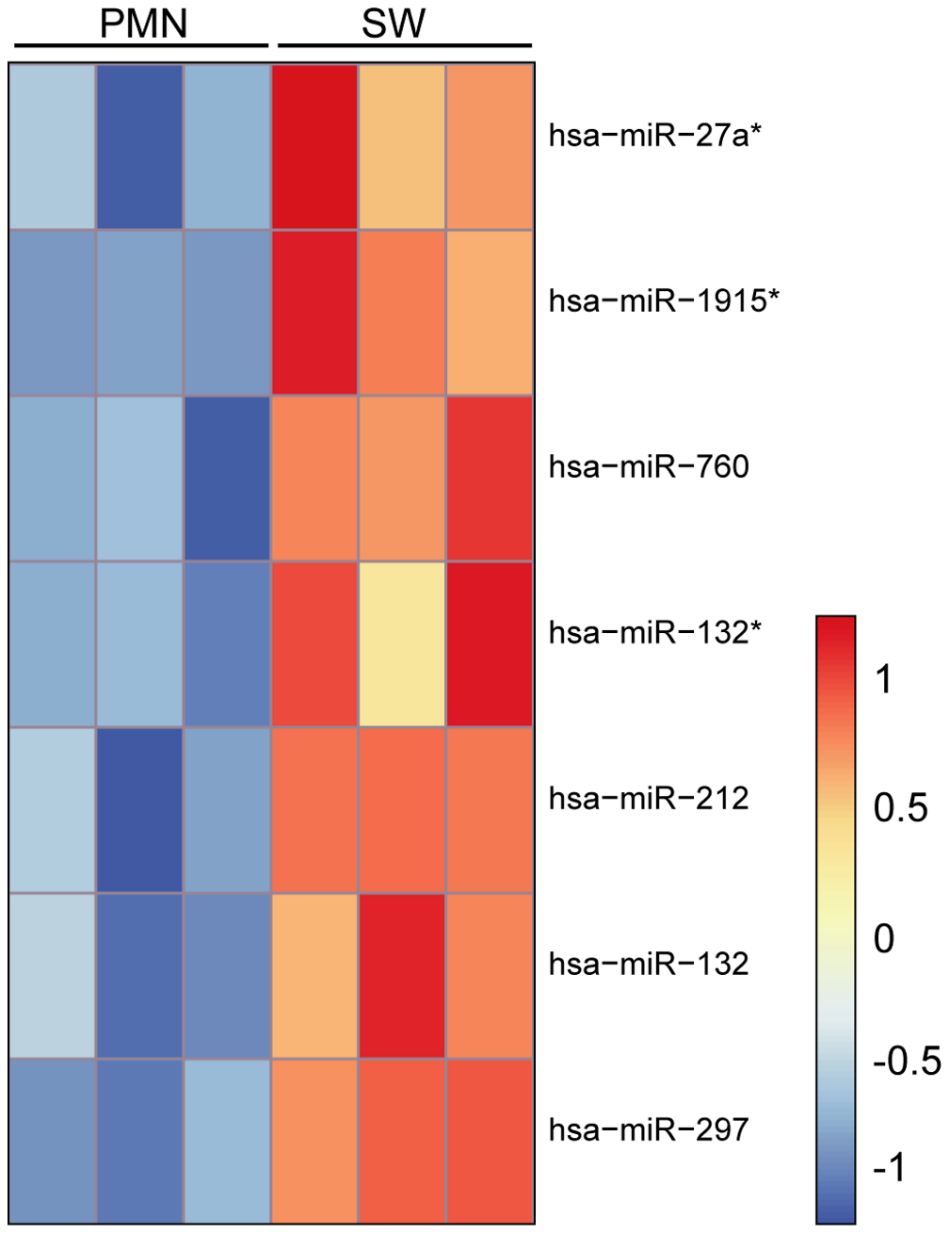
miRNA array analysis of human neutrophils from skin window. Heat map of the seven differentially regulated miRNAs between peripheral blood PMNs and activated neutrophil populations in skin windows. The different miRNAs are listed on the right side of the heatmap and the two different cell populations are marked on the top. Red indicates high expression and blue low expression.

**Figure 5:**
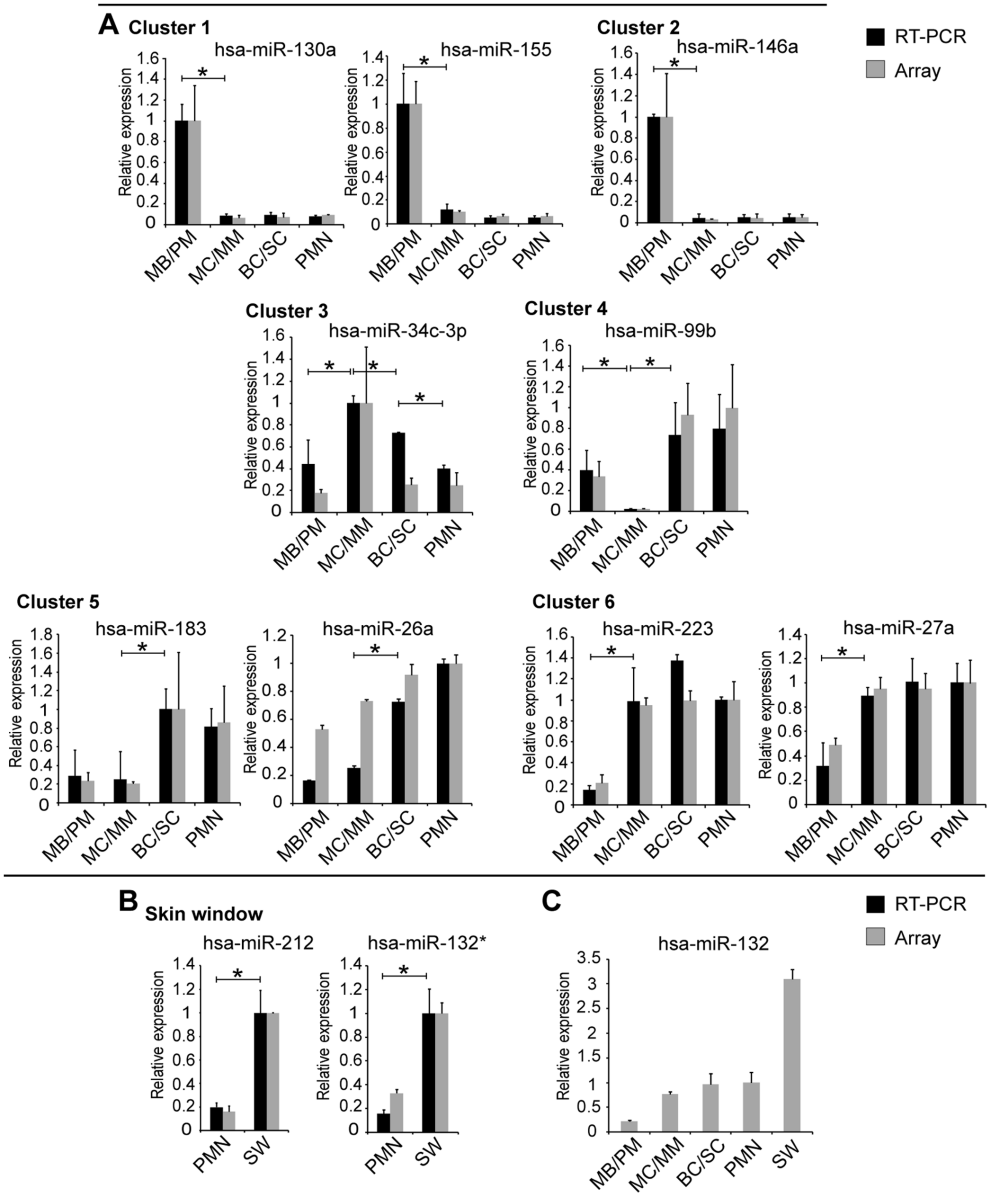
miRNA array data and real-time PCR verification. The miRNA level measured by array analysis is represented by grey columns and the level measured by RT-PCR by black columns. An asterisk indicates a significant difference in expression between two populations (p<0.05) measured by real-time-PCR. A, Real-time-PCR verification of array analysis on selected miRNAs from the six clusters assigned to the miRNAs from bone marrow and peripheral blood PMNs. B, Quantitative real-time PCR verification of array analysis on selected miRNAs from PMNs and skin window PMNs. The relative expression level for each marker is shown relative to the cell population with highest expression, which is assigned the value 1. The real-time PCR data from each donor is measured in triplicates. C, Bar graph showing the schematic miR-132 expression during granulopoiesis and extravasation of PMNs based on array data. Different donors were used for the two different profiling studies. The PMN population has been assigned the value 1 to make the two measurements comparable. Error bars show the difference in expression (SD) between three donors.

Purified RNA from peripheral blood PMNs and PMNs in skin windows were examined for miRNA expression by hybridization to miRNA arrays. We identified seven miRNAs that were differentially expressed in PMNs from peripheral blood compared to neutrophils in skin windows (adjusted p-value <0.05). Interestingly, all seven miRNAs (miR-297, miR-212, miR-1915*, miR-132, miR-27a*, miR-760 and miR-132*) were up-regulated in the skin window neutrophils (Fig. 4 and Table S2). Of special interest is the pattern of miR-132 as it increases from a low expression in MB/PMs to peak expression in neutrophils from skin windows (Fig. 5C).

### Real-time PCR Verification of the Microarray Data on Selected miRNAs

To validate the expression pattern of the differentially expressed miRNAs during normal neutrophil development, we selected representative miRNAs from the six clusters to be analyzed by real-time PCR verified on three to five donors (Fig. 5A). We chose to examine two miRNAs from each of the three large clusters (1, 5, and 6) and one miRNA from each of the three small clusters (2, 3, and 4). The miRNAs were miR-130a and miR-155 (cluster 1), miR-146a (cluster 2), miR-34c-3p (cluster 3), miR-99b (cluster 4), miR-183 and miR-26a (cluster 5), and miR-27a and miR-223 (cluster 6). Except for miR-26a (cluster 5) there was a good correlation between the expression profiles determined by microarray analysis and by real-time PCR. For miR-26a, the level of expression was found to be relative higher in the immature neutrophil precursors when measuring on the array compared to real-time PCR determination. The overall expression pattern does, however, in both cases still show a gradual increase in miR-26a expression level with maturity and the real-time verification shows an even more pronounced difference in expression between the populations. We chose miR-132* and miR-212 among the seven miRNAs up-regulated in exudated neutrophils for validation of the expression pattern between PMNs isolated from blood and the PMNs from skin window (Fig. 5B). Both miRNAs showed good correlation between the expression profiles from the microarray data and the real-time PCR results.

### Prediction of miRNA Targets

The large amount of differentially expressed miRNAs during granulopoiesis and during in vivo exudation does not allow for an in depth analysis of all possible mRNA targets. The data should be considered as a catalogue for further examination of miRNA:mRNA correlations. To illustrate the wide variety of possible mRNA targets, we performed a prediction analysis for the miRNAs we selected for real-time analysis. TargetScan and Miranda algorithms were used for the analysis. Tables 1 and 2 list potential mRNA targets that could be of importance for granulopoiesis and *in vivo* exudation.

**Table 1:** Predicted targets for selected differentially regulated miRNAs in granulopoiesis.

miRNA	Predicted Targets
**hsa-miR-130a**	Both Targetscan and miRanda : MYB, SMAD4, CSF1, TGFBR2, MYBL1, CBFB, PTEN, HOXA5, IGF1, CREB1
	Targetscan: ACVR1, MLL3, CDK19, SMAD5, MAP3K12, TGFBR1, MAPK1, STAT3, E2F2.
	miRanda: IL7, TNF, IL22, IL15, CYBB, E2F7, STAT3, SMAD5, TLR7, MLL4, IL16, TGFBR3, MAP2K5, MAPK6, SMAD7, TAB1, E2F8, MEIS1, AKT3, MAP3K14, MLL5, IL26.
**hsa-miR-155**	Both Targetscan and miRanda: MYB, CEBPB, SP1, MAP3K14, MYBL1, SPI1, CUX1, E2F2, MEIS1, SP3, TAB2
	Targetscan: SMAD2, MAP3K10, TAB2, TRAF3, TBRG1,MYD88
	miRanda: FOS, MLL5, E2F5, E2F3, SP8, SMAD1, IL7, TGFA, OLFM4, GFI1, SMAD6, ELF1, EGFR, SMAD5, E2F2, E2F7, CDK7, MAP3K8, HOXA3, DEFA5, IL23R, JAK2, GATA3, CBFB, AKT2, IL13, TGFBR2, SP2, CYBB, CDK2, PTEN, CEBPD, STAT1, IL18R1, MAPK1, IL12A.
**hsa-miR-146a**	Both Targetscan and miRanda: TRAF6, IRAK1, SMAD4, MYBL1, NOTCH2, SP8.
	Targetscan: CUX1
	miRanda: TLR2, RUNX1T1, MLL5, TAB2, STAT1, CDK9, TGFBR1, RARA,TLR4, CDK1, RUNX1, IL6R, SP3, IL23R, FAS, MEIS1, IL3.
**hsa-miR-34c-3p**	Both Targetscan and miRanda: MAP3K2, FLT3, RUNX1T1.
	Targetscan: CDK17, PDGFB.
	miRanda: HOXA13, IL23R, CREB1, AKT3, HOXA2, IL1A, MAP4K5, FLT1, CYBB, TRAF5, CDK7, MMP8, GATA3, FOXP2, VCAM1, GFI1, ACTR3, IRAK4, CD34, MAP3K9, OLFM4, SP3, IL3RA, TAB2, PTPN9, IL15, IL19, CDK13, NOTCH1, CBFB, CSF1, CUX1, SMAD4, E2F2, F2F5, E2F7.
**hsa-miR-99b**	Both Targetscan and miRanda: MTOR, HOXA1.
	Targetscan: NOX4, IGFR1.
	MicroRNA.org: CYBB, MLL3, FAS, PIK3R3, CDK7.
**hsa-miR-183**	Both Targetscan and miRanda: PTPN4, CUX1.
	Targetscan: TGB1, FOXP1, FOX01, TAB3, MAP3K4, MAP3K13.
	miRanda: SMAD4, PIK3R1, MAPK9, MMP9, SMAD2, NFKB1, TLR8, TAB2, SP7, IL12B, E2F3, EGFR, FAS, CUX2, VCAM1, MTOR, RUNX1, E2F5, MAP2K6, NOTCH2, MYC, PTPN9, MAP3K5, MAPK7, IL8, HOXA5, IL23R, AKT1, MAP2K5, TRAF2, CDK4.
**hsa-miR-26a**	Both Targetscan and miRanda: CBFB, SP3, MYBL1, SP1, CUX1,
	Targetscan: CDC6, CDK8, TAB3, RB1, CREBBP.
	miRanda: IL8R1, MAP3K1, CDK8, TAB2, MAPK6, MAP3K8, MLL3, CREB1, JAK2, MAPK10, VEGFA, CDK12, SMAD6, MAPK13, TGFBR2, STAT2, MAP3K2, IL6, CYBB, HOXD8, SP8, MEIS2, HOXD13, HOXA4, CBFB, TRAF3, SMAD4, CDK1, SMAD5, TGFB2, E2F5, CDK14.
**hsa-miR-223**	Both Targetscan and miRanda: CBFB, SP3, MYBL1, SP1, CUX1,
	Targetscan: TGFBR3, CDK17, E2F1.
	miRanda: EGFR, STAT1, OLFM4, IL3, SMAD1, CDK9, MEIS1, ICAM1, MYC, GFI1, FAS, MAPK4, CDK8, NOTCH2, PTEN, IL23A, IL12B, NOTCH1.
**hsa-miR-27a**	Both Targetscan and miRanda: FOXP2, E2F7, CBFB, RUNX1, RARA, HOXA10, EGFR, TAB3.
	Targetscan: MAP2K4, HOXA5, HOXB8, HOXA13, SMAD9, CREB1, PKIA, TRAF3, SMAD5, CDK18, ITGA2, MLL3, FOXP4, MAPK10, SP6, CDK8, MAPK12.
	miRanda: FAS, MLL3, HOXB5, HOXA9, SMAD9, MAPK13, FOXA3, E2F5, MAPK9, SMAD1, MYB, IRAK4, IL23R, IL16, CSF1, CYBB, PTPN9, IL24, IL12A, MMP7, CDK12, CEBPE, MAP2, CDK18, MLL5, MLL, CEBPG, SMAD4, ICAM2, NOTCH1, PTEN, IL19, E4F1, STAT1.

The table lists the different predicted targets from two different prediction algorithms, miRanda and Targetscan. The targets are listed as predicted by both prediction algorithms or by either one and were selected based on their importance in granulopoiesis and granulocyte function.

**Table 2:** Predicted targets for selected differentially regulated miRNAs between human PMNs and extravasated PMNs in skin window.

miRNA	Predicted Targets
**hsa-miR-212**	Both Targetscan and miRanda: E2F5, MAP3K3, MAPK3, SMAD2, MAPK1IP1L, MEIS1, MAPK1, MYD88, IL1R1, IL1A, IRAK4, IL6R.
	Targetscan: CDK19, MYCBP2, MAPKAP1, CREB5, CUX1.
	MicroRNA.org: EIF2C2, MMP8, RNASEN, SMAD4, STAT1, TGFB1, EIF2C2, MAP2K4, MAP3K8, MAP4KA, MAPK4, MAPKBP1, MLL, MYB.
hsa-miR-132*	Targetscan: Hsa-miR-132* or hsa-miR-132-3p is not accessible on Targetscan.
	miRanda: AKT1, CUX1, E2F2, IL1B, IL6R, IRAK4, MAP2K4, MAP4, MAPK1, MAPK12, MAPK3, MAPK8IP3, MAPKAPK2, MAPKBP1, MPO, TRAF1, TRAF6.
**hsa-miR-132**	Both Targetscan and miRanda: E2F5, IL1A, E2F7, IL1B, IL1BR1, IRAK4, MAP2K4,MAPK1, MAPK3, MAP3K8, MAP3K3, MMP8, MYB, MYD88, RNASEN, RUNX1, SMAD4, TGFBR1, TGFBR2.
	Targetscan: SMAD2, SMAD5, SP8, MEIS1, CUX1, HOXA9.miRanda: HOXA1, IL8, MAP3K1, MAP3K2, MAP3K4, MAP3K5, MAP3K7, MAP3K9,TGFB1, TGFBR3, TLR4.

The table lists the different predicted targets from two different prediction algorithms, miRanda and Targetscan. The targets are listed as predicted by both prediction algorithms or by either one. The targets listed are selected out of the importance in granulocyte function.

### 3′ UTR Analysis of Selected mRNAs Important in Granulopoiesis and in vivo Exudation

To demonstrate a possible role of miRNAs in coordinated regulation of cellular processes important for proper development and function of the neutrophil granulocyte, we decided to examine the relationship between differentially expressed miRNAs and essential transcription factors (RUNX1, C/EBP-ε, and PU.1 [6]) and cell cycle proteins (CDK2, CDK4, CDK6, and p27kip1 [2]) known to be temporally expressed during neutrophil bone marrow development. In addition, we looked at pro-apoptotic proteins (APAF, CASP8, and FADD) found to be repressed in skin window PMNs [7]. The 3′ UTRs of the mRNAs encoding these proteins were analyzed with the miRWalk software. We selected miRNAs predicted by four or more algorithms and containing a seed sequence of minimum 7 nucleotides. Those that are differentially expressed in our arrays are listed in tables 3 and 4.

**Table 3:** Predicted miRNAs targeting the 3′UTR of selected mRNAs important for granulopoiesis.

3′UTR	Cluster	miRNA
*Transcription factors*		
RUNX1	1	hsa-mir-106a, hsa-mir-146b, hsa-mir-17, hsa-mir-181d, hsa-mir-18a, hsa-mir-18b, hsa-mir-20b, hsa-mir-422a, hsa-mir-584, hsa-mir-874
	2	hsa-miR-363, hsa-mir-20b*
	3	–
	4	hsa-miR-7
	5	hsa-mir-182, hsa-mir-185, hsa-mir-192, hsa-mir-194-1, hsa-mir-200c, hsa-mir-22, hsa-mir-22*,hsa-mir-28, hsa-mir-30e, hsa-mir-338-3p, hsa-mir-338-5p, hsa-mir-504, hsa-mir-628, hsa-mir-769
	6	hsa-mir-148b, hsa-mir-15a, hsa-mir-15b, hsa-mir-23a, hsa-mir-23b, hsa-mir-27a, hsa-mir-30a, hsa-mir-30c-1, hsa-mir-424, hsa-mir-550-1,hsa-mir-625, hsa-mir-675.
CEBPε	1	hsa-mir-130a, hsa-mir-130b
	2	–
	3	–
	4	–
	5	–
	6	hsa-mir-454
PU1/SPI1	1	hsa-mir-155
	2	hsa-mir-150
	3	–
	4	–
	5	–
	6	hsa-mir-491-5p
*Cell cycle regulators*		
CDK2	1	–
	2	–
	3	–
	4	–
	5	hsa-mir-200c, hsa-mir-29a, hsa-mir-29b
	6	hsa-mir-140-5p
CDK4	1	hsa-mir-486-5p
	2	–
	3	–
	4	–
	5	–
	6	–
CDK6	1	–
	2	–
	3	–
	4	–
	5	hsa-mir-22, hsa-mir-26a
	6	hsa-mir-15a, hsa-mir-26b
P27/CDKN1B	1	hsa-mir-222, hsa-mir-222*, hsa-mir-155, hsa-mir-181b, hsa-mir-181d
	2	hsa-mir-196b, hsa-mir-152
	3	–
	4	–
	5	hsa-mir-194, hsa-mir-200c, hsa-mir-595
	6	hsa-mir-24, hsa-mir-148

The table lists differentially expressed miRNAs from the array data with predicted targets in the 3′UTRs of mRNAs important in granulopoiesis.

**Table 4:** Predicted miRNAs targeting the 3′UTR of selected mRNAs important in extravasated neutrophils.

3′UTR	miRNA
*Apoptosis regulators*	–
APAF1	hsa-mir-132, hsa-mir-212
CASP8	hsa-mir-132, hsa-mir-212
FADD	hsa-mir-132, hsa-mir-212, hsa-mir-760

The table lists differentially expressed miRNAs with predicted targets in the 3′UTRs of mRNAs encoding pro-apoptotic genes.

## Discussion

Synthesis of cellular factors such as cell cycle proteins, transcription factors, granule proteins, and adhesion molecules is strictly regulated during the differentiation of neutrophil precursors to mature PMNs. Two major events that occur during granulopoiesis are the transition from the proliferative state to cell cycle arrest and the initiation of terminal differentiation in which the neutrophils prepare themselves to recognize and combat invading microorganisms. The latter involves the up-regulation of receptors for pro-inflammatory cytokines and pathogen-associated molecules (PAMs) as well as the formation of neutrophil granules loaded with bactericidal proteins and enzymes required for migration and killing of microorganisms [8]. Extravasation and migration of PMNs to sites of inflammation also result in major changes in the gene and protein expression profiles. Most notable is the shift towards an anti-apoptotic and pro-angiogenetic primed state of the cell, which is accompanied by an additional up-regulation of receptors modulating the inflammatory process and of chemokines and cytokines to recruit other cells in the inflammatory response [3]. A regulatory role for miRNAs has been demonstrated for several of the above-mentioned processes. In order to describe the dynamics of the miRNA pool during granulopoiesis and extravasation, we performed a miRNA microarray analysis of cells representing three distinct differentiation stages in the bone marrow, peripheral blood PMNs, and extravasated neutrophils from skin windows.

Our data reveal 135 miRNAs that are differentially expressed during human granulocytic development. The majority of these were differentially expressed between the MB/PM and the MC/MM stages. The number of differentially expressed miRNAs decreases with maturity and no difference in miRNA expression was observed between BC/SCs and PMNs (Fig. S2). Only seven miRNAs were found to be differentially expressed between peripheral blood PMNs and skin window PMNs. Previous examination of miRNA expression during granulopoiesis has mainly been based on cell lines and peripheral blood PMNs. One study describes the expression of a subset of miRNAs (compared to this study) in flow sorted bone marrow cells [21] but the analyzed cells contained up to 20% non-neutrophil cells. Furthermore, the study did neither include all stages of neutrophil development in bone marrow nor extravasated neutrophils. We therefore believe that a more comprehensive analysis such as ours is of interest.

The transition from MB/PM to the MC/MM stage during granulopoiesis is characterized by an extensive change in the expression of cell cycle related genes [3] and proteins [2] which govern the transition from the proliferative state to cell cycle arrest. The major change in miRNA expression during granulopoiesis also occurs at this stage. This could indicate that miRNAs are important in the fine-tuning of regulatory proteins in this process. The importance of miRNAs in granulopoiesis is underscored by the dysregulation of neutrophil development in mice with a deleted Dicer1 gene (encodes an essential RNase for miRNA processing) in the GMP compartment [22]. Oligodendrocytes, like neutrophils, undergo terminal differentiation in a process that is partly dependent on the progressive accumulation of the cell cycle inhibitor p27kip1 [2]. Deletion of Dicer1 is also found to distort oligodendrocyte development, which could be re-established by over-expressing miR-219 and miR-338 [23].

High levels of miR-130a, miR-155, and miR-146a were observed in MB/PMs. This was followed by a decrease in expression in more mature cells (cluster 1 and 2 profile, Fig. 2). Potential targets for these miRNAs include transcripts encoding members of the TGF-β signalling pathway such as *TGF-β-receptor 1* and *-2, SMAD2, SMAD4, and SMAD5* (table 1). Stimulation of the TGF-β pathway inhibits the proliferation of granulocytic precursors [24] and the deregulation of this pathway is thus advantageous for cell proliferation. A direct role of miR-130a in inhibition of Smad4 synthesis has been demonstrated in neutrophil precursors [24] and the repression of Smad4 mRNA by miR-146a has likewise been demonstrated in the acute promyelocytic leukemia cell line NB4 [25]. Furthermore, it has been shown that miR-155 targets Smad2 mRNA in the monocytic cell line THP1 [26] as well as Smad5 mRNA in lymphoma cells [27]. Together, this points to an important role of miRNAs in dampening the potential growth-inhibitory effect of TGF-β on neutrophil precursors.

Neutrophil lineage decision and differentiation are largely governed by an intricate interplay between different transcription factors. PU.1 is important in determining whether granulocyte-macrophage progenitors (GMPs) develop into monocytes or neutrophils with high PU.1-levels favouring differentiation towards monocytes [28]. As *PU.1* is a direct target for miR-155 [29] the high expression of miR-155 in immature neutrophil precursors probably reduces the amount of PU.1 produced in these cells [6] (table 3). C/EBP-β has the same expression profile as PU.1 during granulopoiesis [6] and is also repressed by miR-155 [30] indicating that these two proteins might be regulated in the same manner. RUNX1 is important for the commitment of hematopoietic precursors to the neutrophil lineage [31] and the expression of early neutrophil genes encoding e.g. MPO and neutrophil elastase is under the control of RUNX1 [32]. Expression of RUNX1 is restricted to the most immature neutrophils [6] and regulates the transition from cell proliferation to cell cycle arrest [33]. It has been shown in the murine myeloblastic cell line 32Dcl3 that miR-27a down-regulates Runx1 and enhances differentiation of the cells [34]. The expression pattern of miR-27a in human bone marrow with increased levels in MC/MMs and more mature cells (cluster 6) fits a model in which translation from residual Runx1 mRNA in MC/MBs is inhibited by miR-27a to promote cell cycle arrest and the initiation of terminal differentiation. 3′ UTR analysis of *RUNX1* reveals several differentially expressed miRNAs that potentially could target *RUNX1* during granulopoiesis. Some miRNAs have an expression profile similar to the Runx1 protein whose mRNA they are predicted to target (cluster 1 and 2), which could indicate that the miRNAs modulate the levels of translation, but do not block translation to protein. The majority of the miRNAs that potentially target the Runx1 mRNA display the opposite expression profile of the Runx1 protein (cluster 5 and 6), which implies a possible role for a miRNA-mediated block of Runx1 expression in the more mature neutrophil precursors (table 3). C/EBP-α is another key mediator of granulopoiesis [12] and has been shown to induce the transcription of miR-223 in concert with PU.1 and C/EBP-β [35], [36], which both have an expression pattern resembling that of miR-223 [6]. Pulikkan et al. demonstrate that miR-223 plays a crucial role in cell cycle regulation by down-regulating the cell cycle protein E2F1 and thereby aiding in promoting cell cycle arrest [19]. We find a low expression of miR-223 in MB/PMs and high expression levels at later stages of maturation (a cluster 6 profile) which fits this proposed function and regulation.

Progression of cells from a proliferative state to cells incapable of division is a strictly regulated process during granulopoiesis. This progression is associated with the down-regulation of CDK2, -4, and -6 and the up-regulation of p27kip1 in the MC/MM stage [2]. The CDK proteins are only expressed in the immature stages of granulopoiesis [2] and 3′ UTR analysis demonstrates that both *CDK2* and *CDK6* are potentially targeted by several miRNAs with a cluster 5 and 6 profile. Studies have shown that miR-22 over-expression down-regulates CDK6 in human fibroblasts [37] and miR-26a/b over-expression down-regulates CDK6 in human liver and lung cells [38], which in both cases causes cell cycle arrest in the G1 phase.

miR-26a and miR-22 have a cluster 5 profile and miR-26b has a cluster 6 profile and thus could be implicated in miRNA mediated cell cycle regulation in human granulopoiesis. Expression of P27kip1 protein is initiated at the MC/MM stage [2] during granulopoiesis. Several studies have shown miR-222 (cluster 1 profile) to be up-regulated in various types of cancers and that it, by targeting p27kip1, thus relieves the inhibition on cell cycle progression from G1 to S phase and allows the cells to continue to proliferate [39]. Also miR-181b (cluster 1 profile) has been shown to down-regulate p27kip1 in a rat liver stellate cell line HSC-T6 [40]. The profiles of miR-222 and miR181b (Cluster 1) thus fit with a role in suppressing P27kip1 in proliferating granulocyte precursors.

The expression profiles of the miRNAs in cluster 3 and 4 indicate a requirement for a temporary regulation of some proteins at the MC/MM stage. The miRNAs in cluster 3 have the same expression pattern as the granulocyte-specific transcription factor C/EBP-ε with specific up-regulation in the MC/MM population [6]. This transcription factor is essential for the expression of genes encoding proteins localized in specific granules and plays an important role in the cessation of cell division at the myelocyte stage and further progress of differentiation [41]. The regulation of C/EBP-ε expression is not yet elucidated, but the miRNAs in cluster 3 could possibly be regulated by the same mechanism(s). The miRNAs in cluster 4 have the exact opposite expression pattern with distinct low expression only in the MC/MM population and could potentially act as regulators of C/EBP-ε protein production during granulopoiesis [6].

Performing 3′ UTR analysis, we only identified three miRNAs that could potentially target the C/EBP-ε mRNA. Two of these have a cluster 1 expression pattern and one has a cluster 6 expression pattern, which could indicate that these miRNAs contribute to fine-tuning of the temporal expression pattern of C/EBP-ε in MC/MM cells.

The function of granulocytes is to identify and destroy invading pathogens. It seems teleologically wise to avoid activation of the neutrophil before it is fully equipped to kill microorganisms. One way of controlling this is to down-regulate intracellular signaling molecules mediating the innate immune response in the immature neutrophil. The IL-1/Toll-like receptor pathway plays an important role in this process [42] and the expression of several of its intracellular signaling molecules is regulated by miRNAs [42]. It has been demonstrated that the transcripts for *TRAF6* and *IRAK1* are targeted by miR-146a [43] and that expression of *MYD88* and *TAB2* are regulated by miR-155 [44], [45]. The high expression of miR-155 and miR-146a in MB/PMs (Fig. 5) could thus participate in the down-regulation of this signalling pathway to prevent a premature immune response.

When comparing the array data from PMNs and skin window PMNs, we found seven differentially regulated miRNAs (miR-297, miR-212, miR-132, miR-132*, miR-1915*, miR-760, and miR-27a*) that were all up-regulated in the extravasated neutrophils. miR-132 is the only miRNA differentially expressed both during granulopoiesis and in skin window PMNs, with a cluster 6 expression pattern and an additional increase in expression in activated neutrophils. Interestingly, miR-212 and miR-132 are part of the same primary transcript and have the same seed region. Although best described in neuronal development, miR-212/132 has also been associated with immune regulation. When stimulated with LPS, human macrophages have been shown to up-regulate miR-132 [46] and both miR-132 and miR-212 can regulate the expression of proteins involved in the inflammatory response [47]. In addition, miR-132 has been shown to be up-regulated in the monocyte/macrophage population from patients suffering from rheumatoid arthritis [48], [48,49] and miR-297 has been found to be up-regulated in serum from patients that died from sepsis compared to survivors [49], [50]. Taken together, this indicates that some of the miRNAs up-regulated during extravasation are involved in immunomodulation. Interestingly, when PMNs are stimulated with LPS none of the seven miRNAs up-regulated during extravasation is altered in expression but instead the expression of miR-9 and miR-9* is up-regulated [49]. This indicates that a subtle regulatory system exists in PMNs that modulate only a few miRNAs depending on the nature of the extrinsic signal to which the PMN is exposed. One of the most distinct changes in cellular functions when PMNs migrate to skin lesions is the down-regulation of pro-apoptotic genes and up-regulation of anti-apoptotic genes [7]. 3′ UTR analysis of the mRNAs encoding the pro-apoptotic proteins APAF1, CASP8 and FADD reveals possible targets for miR-212, miR-132, and miR-760, which are all up-regulated in exudated neutrophils compared to peripheral blood PMNs (table 4). So far, none of these proteins have been described to be regulated by miRNAs and further studies are needed to establish whether these miRs regulate protein expression.

The miRNA expression patterns described in the present paper indicate that miRNAs play a role in the regulation of protein synthesis during granulopoiesis and activation of neutrophils following extravasation. It is also possible that miRNAs play a role in the down-regulation of the immune response after an inflammatory response. Since the data represent the miRNA profiles during granulopoiesis in healthy human bone marrow, this characterization is more reliable than those obtained from *in vitro* differentiation of myeloid cell lines and hematopoietic precursors. Hopefully our data will contribute to further clarification of the different roles that miRNAs play in the differentiation and function of the neutrophil granulocyte in the healthy individual and thereby help to identify dysregulated miRNAs in malignant diseases such as AML.

## Supporting Information

Figure S1 **MDS plot of the four populations in granulopoiesis.** MDS plot of the miRNA expression in the three bone marrow populations and peripheral blood PMNs. Black squares represent the MB/PM populations, the grey bullets the MC/MM populations, the black bullets the BC/SC populations, and the grey squares the PMN populations.
(TIF)

Figure S2 **Venn diagram illustrating miRNA changes between the four populations in granulopoiesis.** Venn diagram illustrating the number of miRNAs differentially expressed between MB/PMs, MC/MMs, BC/SCs, and PMNs (adjusted p<0.05).
(TIF)

Table S1 **Fold Change (FC) values between the four different populations in granulopoiesis.** Table showing FC values between the MB/PM and MC/MM, MC/MM and BC/SC, and BC/SC and PMNs populations of cells and adjusted p-values from the ANOVA analysis for each of the 135 miRNAs. The miRNAs are grouped according to the different clusters.
(DOCX)

Table S2 **Fold Change (FC) values between peripheral blood PMNs and extravasated PMNs in skin window.** Table listing the FC values and the adjusted p-values for the seven differentially regulated miRNAs between peripheral blood PMNs and activated neutrophils in skin windows (SW).
(DOCX)
