# Supplementary figures and images for: MicroRNA Profiling in Human Neutrophils during Bone Marrow Granulopoiesis and In Vivo Exudation
Source: PLoS One. 2013 Mar 12;8(3):e58454. doi: 10.1371/journal.pone.0058454 (PMC3595296; doi:10.1371/journal.pone.0058454)

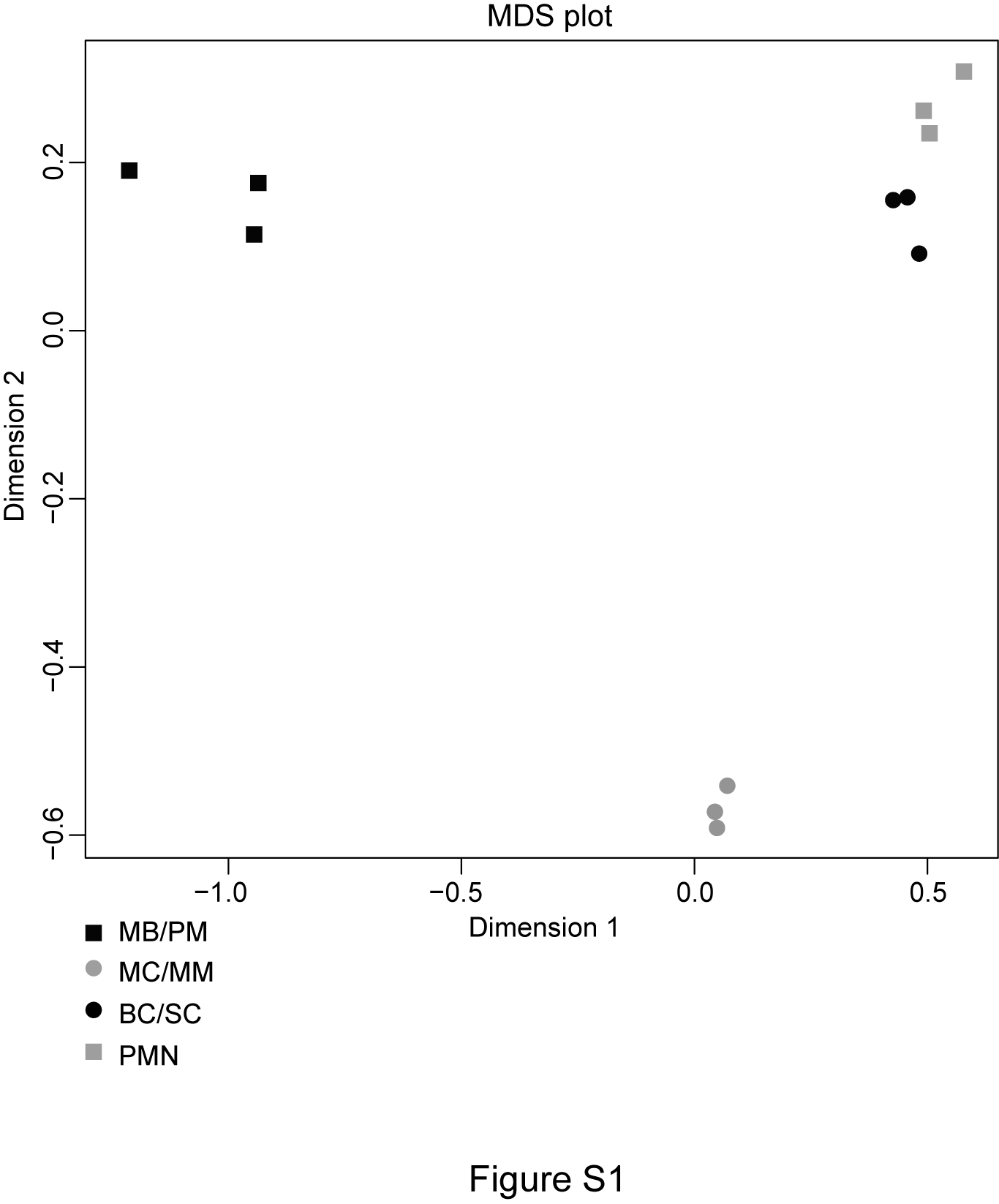

Supplement: Figure S1 — MDS plot of the four populations in granulopoiesis. MDS plot of the miRNA expression in the three bone marrow populations and peripheral blood PMNs. Black squares represent the MB/PM populations, the grey bullets the MC/MM populations, the black bullets the BC/SC populations, and the grey squares the PMN populations. (TIF) [file pone.0058454.s001.tif]

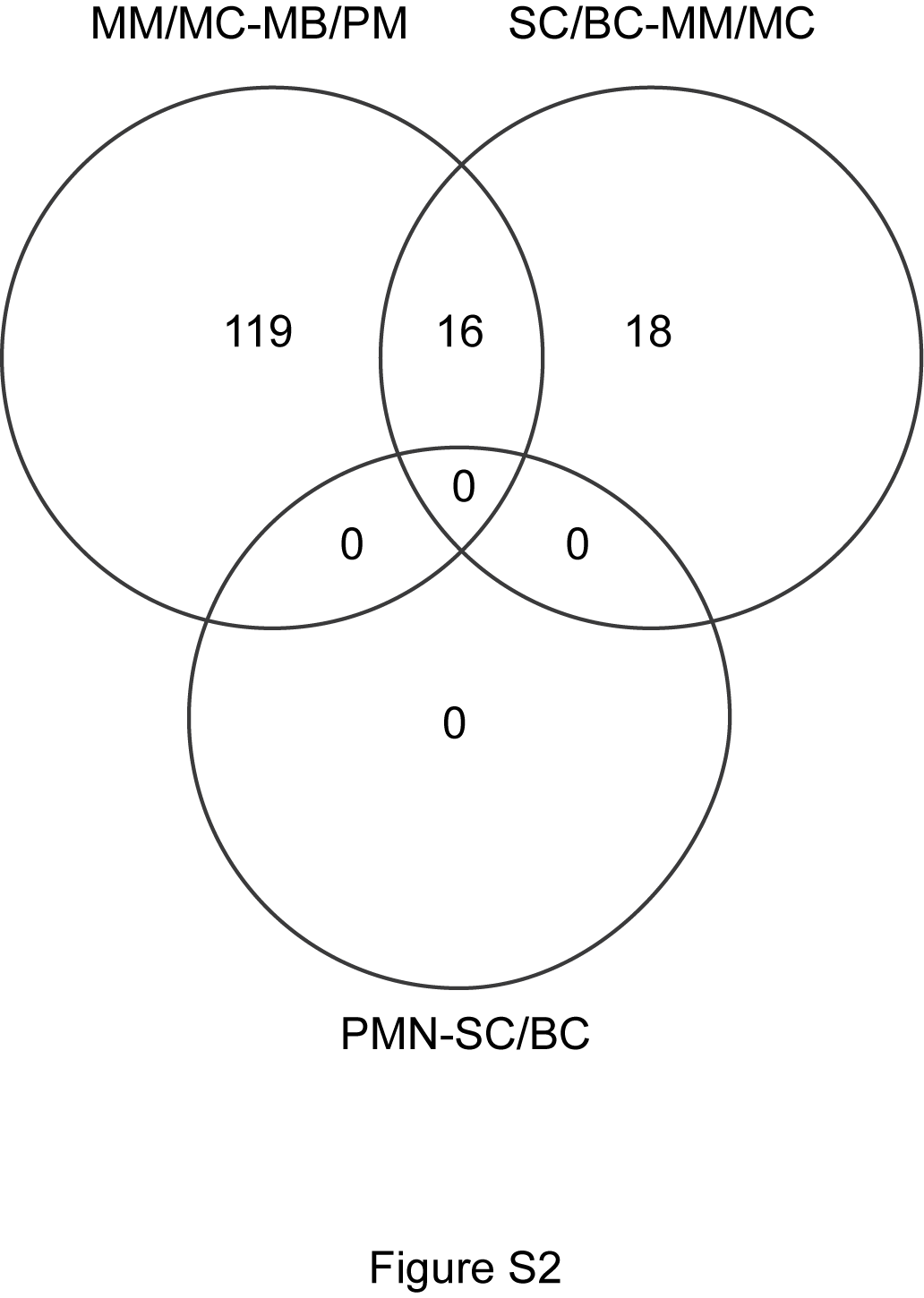

Supplement: Figure S2 — Venn diagram illustrating miRNA changes between the four populations in granulopoiesis. Venn diagram illustrating the number of miRNAs differentially expressed between MB/PMs, MC/MMs, BC/SCs, and PMNs (adjusted p<0.05). (TIF) [file pone.0058454.s002.tif]
