# Supplementary material for: MicroRNA Profiling in Human Neutrophils during Bone Marrow Granulopoiesis and In Vivo Exudation
Source: PLoS One. 2013 Mar 12;8(3):e58454. doi: 10.1371/journal.pone.0058454 (PMC3595296; doi:10.1371/journal.pone.0058454)
Supplement: Table S1 — Fold Change (FC) values between the four different populations in granulopoiesis. Table showing FC values between the MB/PM and MC/MM, MC/MM and BC/SC, and BC/SC and PMNs populations of cells and adjusted p-values from the ANOVA analysis for each of the 135 miRNAs. The miRNAs are grouped according to the different clusters. (DOCX) [file pone.0058454.s003.docx]

**TABLE S1: Foldchange between the different cell populations during granulopoiesis.**

| **Cluster** | **miRNA** | **Fold change** | **Fold change** | **Fold change** | **P-value** |
| --- | --- | --- | --- | --- | --- |
|  |  | **MB/PM- MC/MM** | **MC/MM- SC/BC** | **SC/BC–**  **PMN** |  |
| **1** | hsa-let-7i | 0.01 | -0.93 | -0.08 | 0.00101961 |
|  | hsa-miR-106a | -1.46 | -0.36 | -0.15 | 5.98E-06 |
|  | hsa-miR-10a | -4.29 | 0.12 | -0.04 | 3.16E-06 |
|  | hsa-miR-126 | -5.70 | 0.09 | -0.17 | 1.27E-06 |
|  | hsa-miR-1275 | -0.69 | -2.50 | 0.08 | 2.34E-07 |
|  | hsa-miR-1308 | -0.74 | -2.43 | -0.72 | 0.000601765 |
|  | hsa-miR-130a | -3.83 | -0.04 | 0.50 | 0.000130412 |
|  | hsa-miR-130b | -0.61 | -0.32 | -0.06 | 0.003337054 |
|  | hsa-miR-146b-3p | -1.11 | 0.05 | 0.05 | 0.004588111 |
|  | hsa-miR-151-3p | -4.64 | 0.01 | 0.02 | 3.16E-06 |
|  | hsa-miR-155 | -3.33 | -0.64 | -0.03 | 3.52E-06 |
|  | hsa-miR-17 | -1.39 | -0.36 | -0.16 | 3.37E-06 |
|  | hsa-miR-181a-2-star | -1.27 | -0.15 | -0.24 | 0.00265117 |
|  | hsa-miR-181a-star | -3.09 | 0.35 | -0.66 | 0.001278566 |
|  | hsa-miR-181b | -2.77 | -1.20 | -0.28 | 7.80E-07 |
|  | hsa-miR-181d | -1.48 | -0.95 | -0.05 | 0.00101961 |
|  | hsa-miR-1826 | -0.17 | -0.59 | -0.33 | 0.002611527 |
|  | hsa-miR-18a | -1.11 | -0.76 | -0.10 | 2.98E-05 |
|  | hsa-miR-18b | -1.33 | -0.41 | 0.01 | 6.77E-05 |
|  | hsa-miR-193b | -2.27 | -0.11 | -0.24 | 0.003650737 |
|  | hsa-miR-1979 | -1.29 | -1.44 | -0.58 | 0.000503474 |
|  | hsa-miR-19b | -0.73 | -0.78 | -0.11 | 6.49E-05 |
|  | hsa-miR-20a | -1.39 | -0.53 | -0.10 | 2.51E-05 |
|  | hsa-miR-20b | -1.59 | -0.61 | -0.14 | 0.000130412 |
|  | hsa-miR-222 | -0.78 | -0.16 | -0.02 | 0.006048798 |
|  | hsa-miR-222-star | -1.35 | -0.09 | -0.11 | 5.56E-05 |
|  | hsa-miR-3175 | -1.78 | -0.46 | -0.28 | 0.00018837 |
|  | hsa-miR-378c | -1.00 | -0.44 | 0.04 | 0.004165423 |
|  | hsa-miR-422a | -1.69 | -0.63 | 0.07 | 0.000135694 |
|  | hsa-miR-486-3p | -4.70 | 0.13 | -0.13 | 1.03E-05 |
|  | hsa-miR-486-5p | -6.98 | 0.98 | -0.09 | 2.43E-05 |
|  | hsa-miR-584 | -1.52 | -0.26 | 0.09 | 0.001183871 |
|  | hsa-miR-671-5p | -0.17 | -1.28 | -0.35 | 0.000740631 |
|  | hsa-miR-874 | -1.53 | -1.37 | 0.22 | 0.00018837 |
|  | hsa-miR-92a | -0.88 | -0.14 | -0.02 | 0.007367105 |
|  | hsa-miR-92a-1-star | -2.40 | -1.51 | 0.84 | 3.16E-06 |

| **2** | hsa-miR-125b | | -4.86 | 0.69 | | | 0.53 | 0.000108123 | |
| --- | --- | --- | --- | --- | --- | --- | --- | --- | --- |
|  | hsa-miR-146a | | -4.72 | 0.21 | | | 0.30 | 5.36E-05 | |
|  | hsa-miR-146b-5p | | -2.86 | 0.33 | | | -0.13 | 0.000503474 | |
|  | hsa-miR-150 | | -4.17 | 2.19 | | | 0.33 | 0.000130412 | |
|  | hsa-miR-151-5p | | -6.02 | 0.59 | | | 0.87 | 4.12E-05 | |
|  | hsa-miR-152 | | -2.64 | 0.30 | | | 0.30 | 0.00101961 | |
|  | hsa-miR-196b | | -3.51 | 0.28 | | | 0.75 | 0.005613757 | |
|  | hsa-miR-20b-star | | -2.10 | 0.32 | | | -0.09 | 0.000134271 | |
|  | hsa-miR-31 | | -3.61 | 0.04 | | | 0.68 | 0.001889475 | |
|  | hsa-miR-363 | | -5.40 | -0.52 | | | 0.76 | 3.37E-05 | |
|  | hsa-miR-99a | | -4.57 | 0.24 | | | 0.40 | 6.10E-06 | |
|  |  | |  |  | | |  |  | |
| **3** | | hsa-miR-1280 | 0.53 | | -1.26 | -0.35 | | | 0.003419423 |
|  | | hsa-miR-1469 | 0.57 | | -0.65 | -0.42 | | | 0.005628751 |
|  | | hsa-miR-3193 | 1.92 | | -3.92 | 0.02 | | | 0.008467497 |
|  | | hsa-miR-3196 | 1.23 | | -0.85 | -0.30 | | | 0.000284637 |
|  | | hsa-miR-34c-3p | 2.33 | | -1.88 | -0.14 | | | 0.003558366 |
|  | | hsa-miR-762 | 0.77 | | -0.65 | -0.29 | | | 0.005639997 |

| **4** | hsa-let-7e | -1.03 | 1.75 | 0.41 | 0.007521342 |
| --- | --- | --- | --- | --- | --- |
|  | hsa-miR-125a-5p | -4.26 | 7.05 | 0.61 | 1.71E-05 |
|  | hsa-miR-21 | -0.63 | 1.70 | 0.54 | 0.002103236 |
|  | hsa-miR-7 | -0.46 | 1.56 | 1.22 | 0.003686496 |
|  | hsa-miR-99b | -3.58 | 4.53 | 0.21 | 0.006172025 |

| **5** | hsa-let-7i-star | 0.64 | 1.33 | 0.39 | 0.006922162 |
| --- | --- | --- | --- | --- | --- |
|  | hsa-miR-125a-3p | 0.52 | 2.48 | -0.05 | 0.000176135 |
|  | hsa-miR-1273c | 0.32 | 0.58 | 0.26 | 0.004604391 |
|  | hsa-miR-140-3p | 1.12 | 0.95 | 0.10 | 0.000144521 |
|  | hsa-miR-1471 | 0.21 | 1.16 | -0.29 | 0.003541744 |
|  | hsa-miR-182 | 0.86 | 1.96 | -0.31 | 0.009508015 |
|  | hsa-miR-183 | -0.10 | 2.08 | -0.13 | 0.005588349 |
|  | hsa-miR-185 | 0.39 | 0.66 | 0.06 | 0.002103236 |
|  | hsa-miR-185-star | 2.34 | 0.93 | 0.01 | 5.36E-05 |
|  | hsa-miR-192 | 1.25 | 1.41 | -0.21 | 1.95E-05 |
|  | hsa-miR-194 | 1.44 | 1.78 | 0.13 | 1.71E-05 |
|  | hsa-miR-194-star | 0.82 | 1.29 | 0.56 | 0.001848048 |
|  | hsa-miR-200c | 1.03 | 0.64 | 0.16 | 0.000430363 |
|  | hsa-miR-22 | 0.76 | 0.49 | -0.07 | 0.008898232 |
|  | hsa-miR-22-star | 0.50 | 1.09 | 0.74 | 0.00565806 |
|  | hsa-miR-23a-star | 0.84 | 0.67 | 0.49 | 0.00082845 |
|  | hsa-miR-26a | 0.45 | 0.32 | 0.13 | 0.001309579 |
|  | hsa-miR-28-3p | -0.10 | 1.11 | 0.00 | 0.001369669 |
|  | hsa-miR-28-5p | 0.45 | 0.99 | 0.08 | 0.000209718 |
|  | hsa-miR-29a | -0.14 | 1.89 | 0.10 | 3.37E-05 |
|  | hsa-miR-29b | 0.58 | 1.44 | 0.75 | 0.008780307 |
|  | hsa-miR-29b-1-star | 0.46 | 3.84 | 0.27 | 6.39E-06 |
|  | hsa-miR-30c-1-star | 1.21 | 1.30 | 0.07 | 0.00063099 |
|  | hsa-miR-30e | 0.81 | 0.32 | 0.21 | 0.008898232 |
|  | hsa-miR-3136 | 0.42 | 2.53 | 1.48 | 1.26E-05 |
|  | hsa-miR-338-3p | 0.15 | 1.85 | 0.30 | 0.000306515 |
|  | hsa-miR-338-5p | 2.23 | 3.38 | 0.28 | 3.06E-08 |
|  | hsa-miR-504 | 2.51 | 1.72 | 0.18 | 0.000560014 |
|  | hsa-miR-505-star | 0.87 | 0.77 | 0.22 | 0.000363237 |
|  | hsa-miR-595 | 0.44 | 1.43 | 0.68 | 0.000947494 |
|  | hsa-miR-628-5p | 2.35 | 1.36 | -0.03 | 0.000486848 |
|  | hsa-miR-652 | 0.73 | 0.55 | 0.12 | 0.001577719 |
|  | hsa-miR-664-star | -0.03 | 1.99 | 0.25 | 0.000456601 |
|  | hsa-miR-769-5p | 0.97 | 0.50 | -0.01 | 0.00848155 |

| **6** | hsa-miR-106b-star | 0.70 | 0.14 | -0.05 | 0.008122346 |
| --- | --- | --- | --- | --- | --- |
|  | hsa-miR-1183 | 1.59 | -0.68 | 0.49 | 0.004239481 |
|  | hsa-miR-132 | 1.82 | 0.45 | -0.02 | 1.27E-05 |
|  | hsa-miR-140-5p | 1.78 | 0.02 | 0.01 | 1.03E-05 |
|  | hsa-miR-143 | 2.51 | -0.25 | 0.06 | 1.27E-05 |
|  | hsa-miR-143-star | 1.53 | -0.28 | -0.01 | 0.007521342 |
|  | hsa-miR-145 | 2.57 | -0.52 | -0.22 | 0.003541744 |
|  | hsa-miR-148b | 1.84 | 0.19 | 0.24 | 0.009508015 |
|  | hsa-miR-15a | 0.99 | 0.05 | 0.12 | 0.002922327 |
|  | hsa-miR-15b | 1.28 | 0.45 | 0.04 | 0.005587704 |
|  | hsa-miR-193a-5p | 3.50 | 0.68 | 0.09 | 1.71E-05 |
|  | hsa-miR-197 | 2.60 | 0.58 | 0.14 | 0.000657566 |
|  | hsa-miR-2110 | 1.38 | 0.38 | 0.08 | 0.000425103 |
|  | hsa-miR-2115 | 2.40 | 0.47 | -0.06 | 0.000130412 |
|  | hsa-miR-2115-star | 2.93 | 0.69 | 0.13 | 0.000231223 |
|  | hsa-miR-223 | 2.21 | 0.06 | -0.01 | 0.000105103 |
|  | hsa-miR-23a | 1.08 | -0.07 | 0.01 | 0.004077175 |
|  | hsa-miR-23b | 0.82 | 0.51 | 0.05 | 0.00101961 |
|  | hsa-miR-24 | 0.96 | 0.09 | 0.05 | 0.000130412 |
|  | hsa-miR-24-2-star | 1.76 | 0.12 | 0.15 | 2.55E-05 |
|  | hsa-miR-26b | 1.55 | 0.67 | 0.01 | 0.000829792 |
|  | hsa-miR-26b-star | 2.03 | 0.59 | 0.06 | 0.000900451 |
|  | hsa-miR-27a | 0.96 | -0.01 | 0.06 | 0.00380215 |
|  | hsa-miR-30a | 0.67 | 0.53 | 0.27 | 0.009178733 |
|  | hsa-miR-30c | 1.16 | 0.54 | -0.03 | 0.006030053 |
|  | hsa-miR-424 | 2.65 | -0.19 | 0.75 | 0.008898232 |
|  | hsa-miR-424-star | 1.38 | -0.11 | -0.09 | 0.006920228 |
|  | hsa-miR-4281 | 1.04 | -0.28 | 0.21 | 0.004632115 |
|  | hsa-miR-4286 | 2.08 | 0.32 | -0.03 | 2.16E-05 |
|  | hsa-miR-4317 | 1.36 | 0.28 | 0.02 | 0.003654742 |
|  | hsa-miR-454 | 1.88 | 0.48 | -0.08 | 0.004200712 |
|  | hsa-miR-491-5p | 2.06 | -0.10 | 0.00 | 5.56E-05 |
|  | hsa-miR-542-5p | 2.97 | -0.35 | 0.40 | 0.004034555 |
|  | hsa-miR-550 | 1.84 | 0.01 | 0.16 | 0.001382124 |
|  | hsa-miR-551b-star | 2.10 | 0.84 | -0.24 | 0.001848048 |
|  | hsa-miR-574-5p | 1.19 | -0.11 | 0.23 | 0.003541744 |
|  | hsa-miR-589-star | 1.97 | -0.50 | 0.16 | 0.003419423 |
|  | hsa-miR-625 | 0.73 | 0.31 | 0.14 | 0.005392152 |
|  | hsa-miR-628-3p | 3.09 | 1.28 | 0.16 | 6.36E-08 |
|  | hsa-miR-629 | 1.93 | 1.03 | 0.00 | 3.16E-06 |
|  | hsa-miR-629-star | 2.69 | 0.52 | 0.01 | 0.000130412 |
|  | hsa-miR-675 | 3.04 | -1.74 | -0.10 | 0.000520494 |
|  | hsa-miR-941 | 2.72 | 0.09 | 0.10 | 0.007741364 |
