# Supplementary material for: MicroRNA Profiling in Human Neutrophils during Bone Marrow Granulopoiesis and In Vivo Exudation
Source: PLoS One. 2013 Mar 12;8(3):e58454. doi: 10.1371/journal.pone.0058454 (PMC3595296; doi:10.1371/journal.pone.0058454)
Supplement: Table S2 — Fold Change (FC) values between peripheral blood PMNs and extravasated PMNs in skin window. Table listing the FC values and the adjusted p-values for the seven differentially regulated miRNAs between peripheral blood PMNs and activated neutrophils in skin windows (SW). (DOCX) [file pone.0058454.s004.docx]

**TABLE S2: Foldchange between PMN and skin window neutrophils.**

| **miRNA** |  | **Fold change PMN-SW** | |  | |  | **P-value** |
| --- | --- | --- | --- | --- | --- | --- | --- |
| hsa-miR-297 |  | | 3,05 |  |  | | 0,001098 |
| hsa-miR-212 |  | | 2,69 |  |  | | 0,005108 |
| hsa-miR-27a-star |  | | 1,67 |  |  | | 0,041696 |
| hsa-miR-132 |  | | 1,63 |  |  | | 0,028151 |
| hsa-miR-132-star |  | | 1,56 |  |  | | 0,047448 |
| hsa-miR-1915-star |  | | 1,28 |  |  | | 0,028151 |
| hsa-miR-760 |  | | 1,06 |  |  | | 0,041696 |
